# Nearest-neighbor NMR spectroscopy: categorizing spectral peaks by their adjacent nuclei

**DOI:** 10.1038/s41467-020-19325-4

**Published:** 2020-11-03

**Authors:** Soumya P. Behera, Abhinav Dubey, Wan-Na Chen, Viviane S. De Paula, Meng Zhang, Nikolaos G. Sgourakis, Wolfgang Bermel, Gerhard Wagner, Paul W. Coote, Haribabu Arthanari

**Affiliations:** 1 Department of Biological Chemistry and Molecular Pharmacology, Harvard Medical School, Boston, MA 02115 USA; 2 Department of Cancer Biology, Dana-Farber Cancer Institute, Boston, MA 02215 USA; 3 Department of Chemistry and Biochemistry, University of California, Santa Cruz, CA 95064 USA; 4 Magnetic Resonance Spectroscopy NMR Application, Bruker BioSpin GmbH, 76287 Rheinstetten, Germany

**Keywords:** Structural biology, Solution-state NMR, Quantum mechanics

## Abstract

Methyl-NMR enables atomic-resolution studies of structure and dynamics of large proteins in solution. However, resonance assignment remains challenging. The problem is to combine existing structural informational with sparse distance restraints and search for the most compatible assignment among the permutations. Prior classification of peaks as either from isoleucine, leucine, or valine reduces the search space by many orders of magnitude. However, this is hindered by overlapped leucine and valine frequencies. In contrast, the nearest-neighbor nuclei, coupled to the methyl carbons, resonate in distinct frequency bands. Here, we develop a framework to imprint additional information about passively coupled resonances onto the observed peaks. This depends on simultaneously orchestrating closely spaced bands of resonances along different magnetization trajectories, using principles from control theory. For methyl-NMR, the method is implemented as a modification to the standard fingerprint spectrum (the 2D-HMQC). The amino acid type is immediately apparent in the fingerprint spectrum. There is no additional relaxation loss or an increase in experimental time. The method is validated on biologically relevant proteins. The idea of generating new spectral information using passive, adjacent resonances is applicable to other contexts in NMR spectroscopy.

## Abstract (web summary)

The structure and dynamics of large proteins and complexes can be studied by methyl-NMR but resonance assignment is still challenging. Here, the authors present a NMR method that leverages optimal control pulse design to unambiguously distinguish between Leu and Val using a simple 2D HMQC experiment and they apply it to several proteins including Cas9, interleukin, and human translation initiation factor eIF4a.

## Introduction

NMR spectroscopy enables study of the structure and dynamics of biomolecules in near-native conditions^1–4^. In particular, NMR can probe dynamic events like protein folding, protein–protein, and protein–ligand interactions, and conformational exchange, and characterize lowly populated states^5–8^. However, deriving backbone resonance assignment using traditional triple resonance NMR experiments are restricted to lower molecular weight proteins (e.g., <∼150 kDa). This size limit is due to the rapid relaxation of the magnetization in multidimensional experiments, which is in turn related to slow molecular tumbling of larger molecules.

The advent of ^1^H–^13^C methyl-TROSY experiments and isotope labeling has extended the range of solution NMR to proteins up to 1 MDa in size^9–11^. The CH_3_ protons undergo rapid motion about the methyl axis compared with the rotational tumbling time of proteins. The three chemically equivalent methyl protons give a single peak, which has relatively better sensitivity than backbone resonances^12^. The heteronuclear multiple quantum coherence (HMQC) experiment takes advantage of the cross-correlated relaxation among the methyl protons, known as the methyl-TROSY effect, to transfer the magnetization through slow-relaxing pathways, resulting in narrow linewidths and high intensity peaks^4^. In addition, selective methyl protonation in an otherwise deuterated protein minimizes ^1^H–^1^H dipole-induced relaxation^13–15^. These two advances together make large protein complexes amenable for NMR studies^16^. The amino acids with methyl resonances (Ile, Val, Leu, Ala) typically make up a substantial and well-distributed portion of the protein structure, so these resonances are excellent probes to study dynamics^17^.

Meaningful interpretation of the methyl-NMR data requires that NMR signals for the HMQC experiment have been assigned to sequence specific methyl bearing residue in the protein. For large proteins (>150 kDa), this has been accomplished by mutating individual residues and tracking changes in the spectrum^18^; however, this process is expensive and time consuming. Moreover, specific mutations may interfere with the ability of the protein to fold properly and/or affect expression yields. Recently, developed computational techniques use inter-methyl distance restraints derived from nuclear overhauser effect (NOE) experiments, compared with distances extracted from the protein structure as a means to obtain resonance assignments for methyl residues^19–26^. The central idea is to systematically compare and match candidate assignments for consistency between the known set of inter-methyl distances and NOE distance estimates and choose the best overall match. The search space is typically astronomical; *n* resonances can be matched to *n* methyl groups in *n*! different ways.

Knowledge of the amino-acid type significantly reduces the size of the search space for the computational strategy described above. For instance, compare the matching problem for 30 arbitrary/unclassified amino acids versus jointly matching 15 valines and 15 leucines. The number of combinations, 15! × 15!, is over 100 million times smaller than 30!—a vast difference that can be traded for reduced computational time and complexity, and/or improved reliability of the eventual solution though better coverage of the search space. Fortunately, alanine and isoleucine resonances are easily distinguished by their characteristic spectral frequencies. However, the resonances corresponding to valine and leucine occupy the same spectral space. One way to distinguish leucine from valine peaks in spectrum is by making two different samples using specialized precursors for selective labeling of leucine^27–29^, which is costly and labor intensive, especially for membrane proteins. In addition, unlabeled leucine will add to the proton density, which will affect the relaxation rates of the valines and one has to resort to an expensive deuterated leucine. Similarly, one can also distinguish leucine from valine peaks by preparing an additional sample where valine methyl is labeled with NMR inactive ^12^C rendering those peaks absent from the spectrum. This approach is slightly cheaper than one described before but still it is labor intensive. Another way is to use three-dimensional (or higher) NMR experiments. However, these add extra delays for coherence transfer and encoding of additional dimensions^30–32^. Extra delays result in severe relaxation losses, which can make these approaches unsuitable for large or challenging proteins.

Here, we show that the distinction between methyl resonances corresponding to leucine and valines can be obtained without the drawbacks discussed above using specially designed pulse for selective homonuclear decoupling. We demonstrate the applicability of this selective pulse to distinguish leucine and valines on a variety of proteins.

## Results

### Design of homonuclear decoupling pulse, which selectively affects valine resonances

**Fig. 1:**
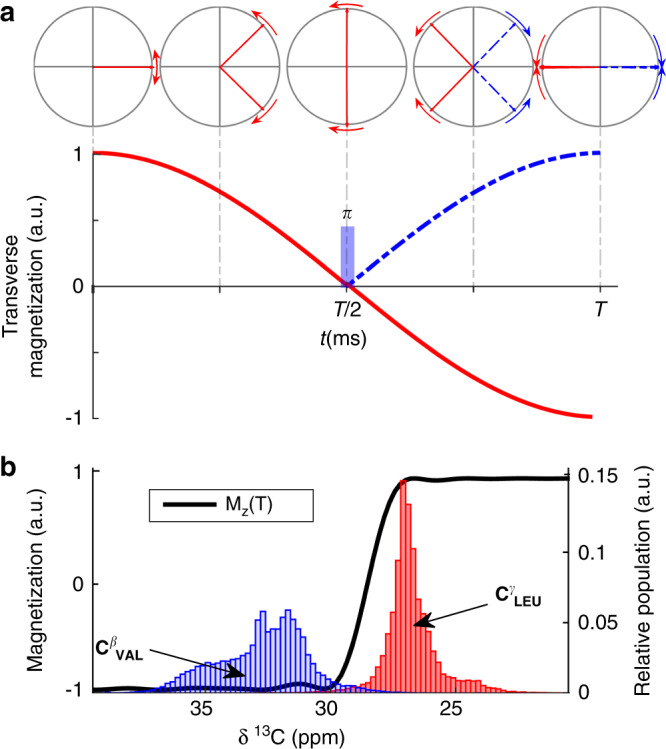
Theory of decoupling using selective inversion to distinguish leucine from valine. **a** Trajectory of the ^13^CH_3_ leucine (red) and valine (blue, dashed) magnetization during a constant time encoding period of duration *T* = 1*/*^*1*^*J*_*CC*_ = 28 ms. Selective decoupling of valine at time *T/*2 refocuses the coupling and produces positive peaks for valine at time *T*. Leucine is not decoupled, and the coupling interaction leads to negative peaks at time *T*. **b** The distributions of C^*β*^ and C^*γ*^ chemical shifts of valine (blue) and leucine (red) are distinct. Assuming an initial state of *I*_*z*_, our decoupling pulse selectively inverts the C^*β*^_Val_, which selectively decouples the valine methyl resonance. The encoding of the methyl-carbon chemical shift continues unaffected during the selective homonuclear decoupling pulse.

In this article, we demonstrate that the leucine and valine signals can be clearly distinguished using a specially designed selective homonuclear decoupling pulse during the indirect ^13^C chemical shift encoding delay. The pulse refocuses the ^13^C^*γ*^_Val_–^13^C^*β*^_Val_ *J*-coupling but does not affect the encoding of the methyl chemical shift frequencies (Fig. 1a). The leucine peaks are not decoupled. As the selective pulse is applied during an existing delay in the experiment, there is no additional loss of transverse relaxation introduced by the pulse compared with a traditional HMQC experiment on the similar isotopically labeled sample. This implies that the molecular weight limit for this approach is the same as for methyl-NMR in general. The idea presented here can be used in both constant-time and real-time indirect encoding schemes, and in many kinds of methyl-NMR experiments including HSQC and HMQC.

Chemical shift data from the BMRB database^33^ show that while distribution of the leucine and valine methyl chemical shifts have considerable overlap, their adjacent nearest neighbor carbon atom (C^*γ*^_Leu_ and C^*β*^_Val_, respectively) have well-separated chemical shifts (Fig. 1b and Supplementary Fig. 1). One way to distinguish methyls of leucine and valine is by transferring magnetization to and from the nearest neighbor. However, that requires additional coherence transfer delays and associated relaxation losses. Instead, selectively inverting C^*β*^_Val_ during the methyl chemical shift encoding leads to a clear distinction between valine and leucine methyl resonances, via selectively refocusing or evolving their one-bond homonuclear *J*-coupling. This difference can be observed as a doublet collapsing into a singlet, in real-time encoding, or a complete sign reversal of only the leucine resonances, in constant time chemical shift encoding (Fig. 1).

Although previous studies have effectively distinguished methyl peaks of threonine and alanine by selective decoupling^34,35^, the small frequency separation between C^*γ*^_Leu_, C^*β*^_Val_, and the encoded methyl region made the approach ineffective in this case; available decoupling pulses were not selective enough to reliably distinguish leucine and valine^30^ and also produced severe spectral artefacts^36^. Modern pulse-design methods, such as optimal control theory, allow precise engineering of pulse selectivity, and dependable Val–Leu distinction is now achievable.

The pulse shape was designed using recently developed homonuclear decoupling techniques from optimal control theory^37^, which permit selective decoupling of closely spaced bands of frequencies without causing any undesirable off-resonance effects, such as Bloch–Siegert shifts^38^. The pulse is applied in the middle of the ^13^C indirect encoding period. During the pulse, the transverse component of the methyl magnetization (for all Ile, Leu, Val, Ala residues) evolve under their intrinsic chemical shift frequencies (Supplementary Fig. 2) for an apparent delay of *T*′ = *aT*, where *T* is the duration of the pulse and *a* (0 < *a* ≤ 1) is a constant chosen during pulse design. The encoding delays on either side of the relatively short pulse can easily absorb an extra (1−*a*)*T* of chemical shift encoding, so that the overall increment is exactly compatible with the Nyquist grid, Fourier transform, and all further downstream data processing. In this case, we determined by numerical exploration that *a* = 0.84 and *T* = 2.3 ms were suitable to design a valine-selective decoupling pulse.

To numerically optimize the pulse, an ensemble of 50 spins with uniformly spaced chemical shifts *ω* in range of [29.2, 44] ppm was sampled to represent the C^*β*^_Val_. An initial state of *ρ*(0) = *I*_*z*_ and a desired final state of *λ*(*T*) = −*I*_*z*_ was set for these spins. Inversion of the passive spins is sufficient for decoupling^37^. To ensure the pulse achieves proper chemical shift encoding in the nearby methyl region, we sampled 130 spins from *ω* ∈ [8.5, 27.5] ppm and alternated the initial state *ρ*(0) between *I*_*x*_ and *I*_*y*_. A desired final state given by *λ*(*T*) = *Uρ*(0)*U*^†^ where *U* = *exp*(−*iaωTI*_*z*_) is set individually for the spins based on their intrinsic chemical shift frequency *ω*. The simultaneous optimization of inversion and encoding behavior ensures that decoupling does not cause any off-resonance effects on the methyl spins. The optimized pulse shape was generated using the toggling frame implementation^37–39^ of the GRAPE (gradient ascent pulse engineering) algorithm^40–44^. The optimization was seeded with a random pulse shape. The algorithm maximizes the agreement between the target state *λ*(*T*) and the simulated final state *ρ*(*T*), by systematically changing the pulse shape using gradient ascent optimization. The resulting radio frequency amplitude and phase, and a simulation of the performance in the methyl chemical shift region are given in Supplementary Fig. 2. The various spins follow highly intricate trajectories during the pulse, but all end up at their respective desired final states at time *T* (Supplementary Fig. 4).

### Testing the designed pulse on maltose-binding protein

**Fig. 2:**
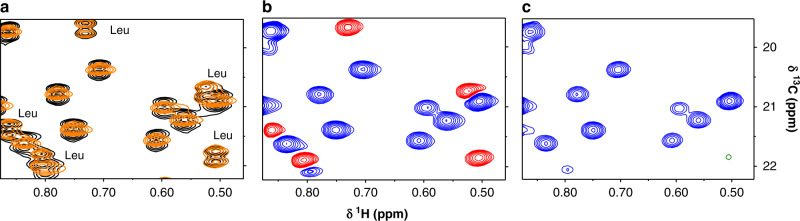
Testing the selective decoupling pulse using real time and constant-time ^1^H–^13^C HMQC on MBP. **a** Overlay of a section of a real-time 2D ^1^H–^13^C methyl HMQC experiment with (orange) and without (black) the selective decoupling pulse on a 500 µM MBP (42.5 kDa) protein. The leucine peaks remain as doublets, whereas the valine peaks collapse into singlets. **b** Constant-time ^1^H–^13^C HMQC experiment with indirect constant-time period set to *T* = 28 ms (1*/*^1^J_CC_) and shaped pulse applied at time *T/*2. Valine resonances are positive (blue) whereas leucine peaks are negative (red). **c** A version of the experiment with constant-time evolution period is set to *T* = 42 ms (3*/*(2 ^*1*^*J*_*CC*_)) completely eliminates the leucine peaks from the spectrum with positive valine peaks remaining (blue). Full spectra are in Supplementary Fig. 5.

**Fig. 3:**
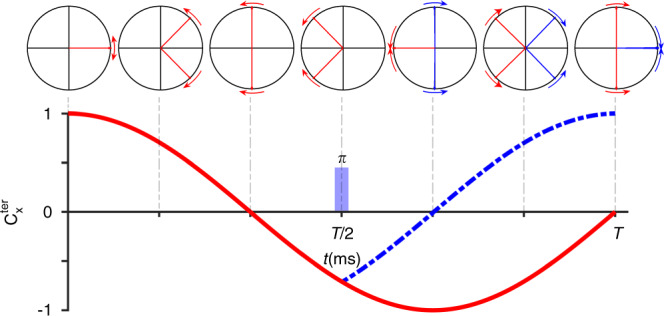
Schematic explaining disappearance of isoleucine and leucine peaks when the constant-time duration is set to T = 3/(2 ^1^J_CC_). The simulation shows the transverse projection of the ^13^CH_3_ Leu (red), Val (blue) magnetization vector, with arrows showing the sense of rotation with respect to the intrinsic chemical shift frequency. The resulting ^13^CH_3_-x magnetization for Leu (red), Val (blue, dashed) are shown below. The constant-time evolution period *T* is set to 42 ms (3*/*(2 ^*1*^*J*_*CC*_)). The shaped pulse at the *T*/*2* selectively decouples C^*γ*^_Val_ from C^*β*^_Val_, resulting in the appearance of the valine peaks. The disappearance of isoleucine and leucine peaks in the spectrum is due to the cancelling out of the anti-phase term arising because of the ^13^C–^13^C spin coupling.

We tested this method on selective ILV-methyl labeled and otherwise deuterated 42 kDa maltose-binding protein (MBP). A high-resolution HMQC (<35 Hz resolution in ^13^C dimension) has doublets owing to the evolution of ^1^J_CC_ homonuclear coupling. This coupling was selectively refocused for valine methyl by using the selective pulse described above (Fig. 2a). The increased crowding owing to peak splitting may be undesirable for large proteins. In such cases, distinction can be achieved by imparting an opposite sign to valine versus leucine methyl resonances by using constant time encoding of methyl chemical shifts (Fig. 1a). The spectra in Fig. 2b, c were recorded using a constant-time two-dimensional (2D) HMQC pulse sequence (Supplementary Fig. 3) with constant-time *T* set as 1*/*^*1*^*J*_*CC*_ and 3*/*(2 ^*1*^*J*_*CC*_), respectively. The latter spectrum has only valine methyl peaks, enabling identification of any leucine-valine overlapping peaks. The theory explaining the disappearance of leucine methyls when *T* = 3*/*(2 ^*1*^*J*_*CC*_) is shown in Fig. 3.

### Validating the designed pulse on other challenging proteins

**Fig. 4:**
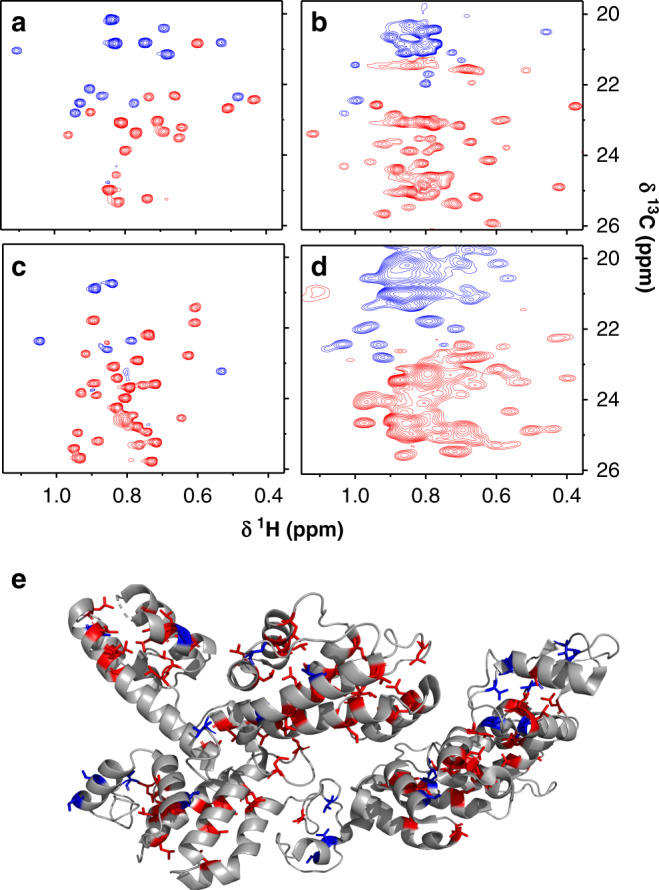
Application of the valine-selective decoupling pulse on biologically relevant proteins. Constant-time 2D ^1^H–^13^C methyl HMQC spectra of four biologically relevant proteins are shown. **a** Cas9 HNH domain (130 µM, 15 kDa); **b** Cas9 REC1-2 domain (250 µM, 52 kDa); **c** Human IL2 (920 µM, 15.4 kDa); **d** eIF4A (50 µM, 46 kDa). Unambiguous distinction between the valine (blue) and leucine (red) resonances is achieved. Only Leu–Val overlapping region of the HMQC spectrum is shown here. The full spectra of these proteins are presented in Supplementary Figs. 6 and 7. **e** Here we show the 3D structure for the REC1-2 domain of Cas9 (PDB ID: 4UN3)^52^ where the valine and leucine residues are highlighted in blue and red, respectively.

We expanded our test set by including range of biologically relevant protein samples and different magnetic field strengths. It should be noted that the same decoupling pulse can be used at different field strengths by linearly scaling the pulse length and power level accordingly. The results observed are as expected on a suite of four proteins, namely the HNH domain of Cas9, the REC1-2 domain of Cas9, human-IL2, and eIF4A (eukaryotic initiation factor A) (Fig. 4, full spectra are shown in Supplementary Figs. 6 and 7). We used the valine methyl-selective decoupling pulse in the SOFAST-HMQC for acquiring spectra on these proteins^45^ to achieve increased sensitivity per unit time. Where assignments are known, our method correctly identifies the valine and leucine peaks. In the absence of assignment, we matched the expected number of peaks for valine and leucine methyl with the positive and negative peaks in the spectrum. The broadened peaks in eIF4A reflect the dynamic nature of protein, which is confirmed in the control spectrum (without selective decoupling pulse). As described before, we can see in an example of Rec1-2 domain of Cas9 structure the valine and leucine methyl are distributed throughout the protein, enabling them to act as NMR probes for different protein domains. Another point to note from Fig. 4e is that valine and leucine are found in close proximity on the surface of the protein. As valine and leucine also overlap in chemical shift space, it is often challenging to derive resonance assignments solely based on distance constraints obtained through NOEs. Thus, the ability to distinguish them by any other NMR experiment, as shown in Fig. 4b is invaluable for obtaining unambiguous methyl assignments.

## Discussion

The method we have presented here can provide amino-acid classification for resonance assignment using a variety of different NMR experiments (notably HMQC in real-time, constant-time and SOFAST). However, the basic principle is easily adapted to related contexts, provided that there is an indirect encoding delay for the carbon methyl resonances for example, depending on choice of constant-time duration and/or phase cycle, either valine or leucine peaks can be selectively removed or moved in the spectrum. We can also selectively remove valine methyl peaks from an HMQC spectrum by taking difference of two spectra recorded with and without the pulse in an interleaved manner. We can selectively shift the resonances of valine methyl to another less crowded part of the spectrum; for example, by using the decoupling pulse only in one of the quadrature components of the indirect acquisition scheme, or only on every second indirect increment^46^. Such tricks can be used to obtain clear distinction between overlapping leucine and valine methyl peaks. This may be useful to alleviate crowding or ambiguity in NOE experiments or in chemical shift perturbation measurements.

The same selective pulse can be used at different magnetic field strengths by proper scaling of pulse duration and pulse power. We acquired the data for MBP on a 750 MHz NMR spectrometer and for other proteins on an 800 MHz NMR spectrometer. In addition, we acquired constant-time HMQC with constant time period set to 28 ms for MBP on a 600 MHz and an 800 MHz NMR spectrometer, with the selective pulse, to demonstrate the pulse applicability across different magnetic field strengths (Supplementary Fig. 8). The pulse duration and power for different magnetic field strengths are listed in Supplementary Table 1.

The method can be used in both real time and constant time versions of the HMQC as shown above. In the real-time experiments, the differentiation can be seen in the peak shape, by the presence or absence of the splitting. In particular, the valine residues will appear as a singlet and the leucine as a doublet. In the constant time implementation, the differentiation is much more dramatic and there is a sign inversion between the leucine and valines peaks. However, the constant-time implementation suffers from relaxation loses when we deal with very large proteins, which can be compensated by additional scans and the use of non-uniform sampling (NUS).

We theoretically estimated transverse relaxation for perdeuterated protein where only CD1 of Ile, CG1 of Val, and CD1 of Leu protonated. We used the following relaxation estimates from a recent review by Schutz et al.^47^. For a protein tumbling with rotational tumbling time 20 ns, the transverse relaxation rate (*R*_*2*_) of CH multiple quantum coherence is estimated to be ~5 Hz. And for this *R*_*2*_ of 5 Hz, the loss of signal height due to constant time encoding relative to real time encoding is 8%. Similarly, for a protein tumbling with rotational tumbling time 180 ns, the transverse relaxation rate (*R*_*2*_) of CH multiple quantum coherence is estimated to be ~42 Hz. And for this *R*_*2*_ = 42 Hz, the loss of signal height due to constant time encoding relative to real time encoding is 46%. To put into perspective of molecular size, 180 ns is the tumbling time of proteasome (650 kDa) at 65 °C. Here the user can employ a real time implementation of the experiment, which will allow to distinguish leucine from valine or use constant time implementation with more scans and additionally use NUS for time savings.

The implementation described here, in either HMQC or HSQC form, can be appended to NOESY experiments; for instance, a 3D NOESY-HSQC or a 4D HMQC-NOESY-HMQC. These types of experiments can be used to distinguish leucine-derived spatial constraints from those originating from valine. Simple, reliable amino-acid determination promises to improve the utility and reliability of existing and emerging methyl-NMR assignment software. Amino-acid selectivity can also be used to simplify and clarify methyl-NMR spectra in a range of experimental contexts.

## Methods

### *Streptococcus pyogenes* Cas9

The coding sequences for Cas9 HNH domain (residues 776–908) and REC1-2 domain (residues 56–505) were synthesized and codon optimized for expression in *Escherichia coli* (Supplementary Table 3). HNH domain was expressed with a C-terminal 6× His tag, and REC1-2 domain was fused with a MBP tag, an N-terminal 6× His and a tobacco etch virus (TEV) protease cleavage site. Each protein was expressed in *E. coli* BL21 (DE3) containing chaperone plasmid pG-KJE8 (TAKARA, 3340) to enhance protein folding. In brief, when cells reached an OD_600_ of ~0.6, isopropyl β-d-1-thiogalactopyranoside (IPTG) was added to a final concentration of 0.5 mM to induce protein expression. Cells were then grown for an additional 18 h at 23 °C. Collected cells were resuspended in lysis buffer (50 mM) Tris pH 7.5, 500 mM NaCl, 5% (v/v) glycerol and 1 mM Tris(2-carboxy-ethyl) phosphine (TCEP) containing an ethylenediaminetetraacetic acid (EDTA)-free protease inhibitor tablet (Roche). The cell suspension was sonicated on ice and clarified by centrifugation at 15,000 × *g* for 30 min. The soluble lysate fraction was bound in batch to nickel-nitrilotriacetic acid (Ni-NTA) agarose (Qiagen). The resin was washed extensively with 20 mM Tris, pH 7.5, 500 mM NaCl, 1 mM TCEP, 10 mM imidazole, and 5% (vol/vol) glycerol, and the bound protein was eluted in 20 mM Tris, pH 7.5, 500 mM NaCl, 1 mM TCEP, 300 mM imidazole, and 5% (vol/vol) glycerol. For REC1-2 domain, the His_10_-MBP affinity tag was removed with His_10_-tagged TEV protease (1 mg of TEV protease was added per 50 mg of fusion protein) during overnight dialysis against 20 mM Tris, pH 7.5, 500 mM NaCl, 1 mM TCEP, and 5% (vol/vol) glycerol. The protein was then flowed over Ni-NTA agarose to remove TEV protease and the cleaved affinity tag and further purified by size-exclusion chromatography on a Superdex 200 16/60 column (GE Healthcare) in 20 mM Tris, pH 7.5, 200 mM KCl, 1 mM TCEP, and 5% (vol/vol) glycerol. For HNH domain, following the Ni-NTA purification, the protein was further purified on a Superdex 200 16/60 column (GE Healthcare) in 20 mM HEPES (pH 7.5) and 80 mM KCl. Protein concentrations were determined using A_280_ measurements on a NanoDrop with extinction coefficients estimated with the ExPASy ProtParam tool.

### Interleukin-2

Codon optimized DNA encoding the human IL2 (Supplementary Table 3) was expressed in *E. coli* BL21 (DE3) competent cells as inclusion bodies. Protein expression was achieved by induction with 1 mM IPTG at an OD_600_ of 0.6 followed by cell growth at 37 °C for 5 h at 200 r.p.m. For in vitro refolding, ~30 mg of inclusion bodies was dropped diluted into 200 mL of refolding buffer (1.1 M guanidine, 6.5 mM cysteamine, 0.65 mM cystamine, 110 mM Tris pH 8.0) at 4 °C while stirring. Refolding proceeded overnight at 4 °C without stirring. The solution was dialyzed into a buffer of 20 mM MES, pH 6.0, 25 mM sodium chloride. Purification of refolded IL2 was performed by cation exchange chromatography with a CAPTO-SP column using a 25 mM to 1 M NaCl gradient in a buffer with 25 mM MES pH 6.0 followed by size-exclusion chromatography on a Superdex 75 column (GE) in 50 mM NaCl, 20 mM sodium phosphate, pH 6.0.

HNH, REC1-2 and hIL-2 were overexpressed in M9 minimal media culture in ^2^H_2_O containing 2 g l^−1 2^H^13^C glucose (Sigma #552151) and 1 g l^−1 15^NH_4_Cl (Sigma #299251). Selective methyl labeling referred to as ILV*, was achieved by the addition of appropriate precursors (ISOTEC Stable Isotope Products (Sigma-Aldrich) as detailed previously^48,49^. The selective labeling of ILV* methyls (Ile ^13^Cδ_1_ only; Leu ^13^Cδ_1_/^13^Cδ_2_; Val ^13^Cγ_1_/^13^Cγ_2_ in an otherwise U-[^15^N, ^13^C, ^2^H] background) was achieved by adding 60 mg l^−1^ 2-ketobutyric acid-^13^C_4_,3,3-^2^H_2_ (Sigma #607541) for Ile and 120 mg l^−1^ 2-keto-3-(methyl-d3)-butyric acid-1,2,3,4-^13^C_4_, 3-2H (Sigma #637858) for Leu/Val 1 h prior to induction. For each uniformly ILV*-labeled protein sample, the concentration and buffer composition are as follows:

- The 0.13 mM HNH domain (15 kDa) in 20 mM HEPES (pH 7.5), 80 mM KCl, 0.01% NaN3, 10% D_2_O.
- The 0.25 mM REC1-2 domain (52 kDa) in 20 mM HEPES (pH 7.5), 200 mM KCl, 5% deuterated glycerol-d8, 1 mM TCEP, 0.01% NaN_3_, 10% D_2_O.
- The 0.9 mM hIL-2 (15 kDa) in 20 mM sodium phosphate (pH 6.0), 50 mM NaCl, 0.01% NaN_3_, 10% D_2_O.

### MBP

Several single colonies of BL21 (DE3) cells carrying MBP encoding plasmid was grown overnight at 37 °C in 10 ml LB media with 100% D_2_O. The cells were pelleted and resuspended in 10 mL M9 medium in D_2_O containing 2 g of *U*[^13^C, ^2^H] labeled glucose and *U*[^15^*N*] labeled NH_4_Cl and grown for 12 hours at 37 °C. The culture was expended to 100 mL in the same medium and continue to grow at 37 °C. When OD_600_ reach 0.4, 70 mg/l of *α*-ketobutyric acid (CDLM-4611 from Cambridge Isotope Laboratories) and 120 mg/l of *α*-ketoisovaleric acid (CDLM-4418 from Cambridge Isotope Laboratories) was added. Both these precursors were uniformly ^13^C labeled and deuterated, with the exception of the methyl group, which was protonated. The protein expression was conducted for overnight at 18 °C after induced with 1 mM IPTG at OD_600_ 0.6–0.8. The cells were harvested and lysed using sonication. The lysate was passed through immobilized amylose beads and was eluted using 10 mM maltose in 50 mM Tris-HCl, pH 8.0. The eluted protein was buffer exchanged into 10 mM HEPES, 1 mM EDTA, pH 6.5. The amides were back exchanged with protons by incubating it with 1 M urea for 24 h at 37 °C. This was followed by buffer exchange to NMR buffer and further purified on size-exclusion column (Superdex 75 10/300 GL). The final sample was prepared by concentrating MBP to 0.5 mM into NMR buffer (1 mM EDTA, 2 mM *β*-cyclodextrin, 10 mM HEPES, pH 6.5).

### eIF4A

The human translation initiation factor eIF4A (46 kDa) was expressed using a N-terminal His_6_-GB1-tag followed by TEV cleavage site in *E. coli*. Protein expression and isotope labeling were performed as described above for the case of MBP, except that 500 mL cell culture was used instead. eIF4A was purified by loading the lyse in buffer A (25 mM sodium phosphate, pH 7.5, 300 mM NaCl, 2 mM beta mercaptoethanol, 5% glycerol) plus 10 mM imidazole to Ni-NTA resin, washed with buffer A containing 30 mM imidazole and eluted with buffer A plus 300 mM imidazole. The elution was dialyzed in presence of His_6_-TEV protease at 1:40 (w:w) ratio overnight against 4 L buffer A. When the cleavage of GB1-tag is completed, the reaction mixture was purified by running though fresh Ni-NTA resin. Flow-through containing eIF4A was collected, concentrated and further polished using superdex 75 10/300 GL column equilibrated in buffer B (20 mM Tris-HCl at pH 7.5, 150 mM NaCl, 5 mM DTT). The final NMR sample is prepared at 50 μM concentration in buffer B with 10% D_2_O.

### NMR data acquisition and analysis

All NMR experiments were acquired on Bruker NMR machines operating at 600 MHz, 750 MHz, or 800 MHz ^1^H Larmor frequency and equipped with cryogenically cooled triple resonance 5 mm probes. The samples were doped with 5% D_2_O for the purpose of locking. 2D ^1^H-^13^C HMQC or ^1^H-^13^C SOFAST-HMQC NMR experiments were recorded on different protein samples with the acquisition parameters provided in Supplementary Table 2. The spectra were acquired using Topspin 3 acquisition software from Bruker. They were processed using NMRPipe^50^ and analyzed using in house MATLAB scripts and CCPNMR^51^. The selective homonuclear decoupling pulse, real-time and constant-time version of HMQC pulse sequence used in this manuscript is provided as Supplementary Data 1–3, respectively.

### Reporting summary

Further information on research design is available in the Nature Research Reporting Summary linked to this article.

## Supplementary information

Supplementary Information

Description of Additional Supplementary Files

Supplementary Data 1

Supplementary Data 2

Supplementary Data 3

Reporting Summary

## Data Availability

The shaped file used in this manuscript is provided as Supplementary Data 1. NMR data sets are available from the corresponding authors upon reasonable request. Pulse sequences, shaped pulse files, and related parameter sets, along with detailed instructions can be downloaded from the lab website http://artlab.dana-farber.org/downloads.html.

## Code availability

Pulse sequences are provided as Supplementary Data 2 and 3.
