# Supplementary material for: Nearest-neighbor NMR spectroscopy: categorizing spectral peaks by their adjacent nuclei
Source: Nat Commun. 2020 Nov 3;11:5547. doi: 10.1038/s41467-020-19325-4 (PMC7642304; doi:10.1038/s41467-020-19325-4)
Supplement: Supplementary file 1 — Supplementary Information [file 41467_2020_19325_MOESM1_ESM.pdf]

Supplementary Information

Nearest-neighbour NMR spectroscopy: categorizing spectral peaks by their adjacent nuclei

Soumya P. Behera,[1]† Abhinav Dubey,[1,2]† Wan-Na Chen,[1] Viviane S. De Paula,[3] Meng Zhang,[1] Nikolaos G. Sgourakis,[3] Wolfgang Bermel,[4] Gerhard Wagner,[1] Paul W. Coote,[1,2]* Haribabu Arthanari [1,2]*

[1]Department of Biological Chemistry and Molecular Pharmacology, Harvard Medical School, Boston MA 02115, USA
[2]Department of Cancer Biology, Dana-Farber Cancer Institute, Boston, MA 02215, USA
[3]Department of Chemistry and Biochemistry, University of California Santa Cruz, CA 95064, USA
[4]Magnetic Resonance Spectroscopy NMR Application, Bruker BioSpin GmbH, 76287 Rheinstetten, Germany

*Co-corresponding authors. Email: paul_coote@hms.harvard.edu
hari@hms.harvard.edu
† Contributed equally

Supplementary Fig. 1. Chemical shift distribution of the methyl and adjacent bonded carbon resonances.
Supplementary Fig. 2. Properties of the valine-selective decoupling pulse designed using optimal control theory.
Supplementary Fig. 3. Pulse sequence diagram for constant time SOFAST-HMQC.
Supplementary Fig. 4. Simulation of the magnetization trajectory for selected chemical shifts under the influence of the shaped pulse.
Supplementary Fig. 5. ^{1}H–^{13}C HMQC spectra of MBP with various implementations of the valine specific decoupling pulse.
Supplementary Fig. 6. ^{1}H–^{13}C HMQC spectra of human IL-2 and Cas9 HNH domain.
Supplementary Fig. 7. ^{1}H–^{13}C HMQC spectra of Cas9 Rec1-2 domain and eIF4A.
Supplementary Fig. 8. Applicability of the selective pulse at different magnetic field strengths.
Supplementary Table 1. Scaling of pulse duration and pulse power with magnetic field.
Supplementary Table 2. Two dimensional ^{1}H-^{13}C HMQC NMR experiment acquisition parameters for proteins used in this study.
Supplementary Table 3. Sequences of codon optimized genes for *E. coli* expression.

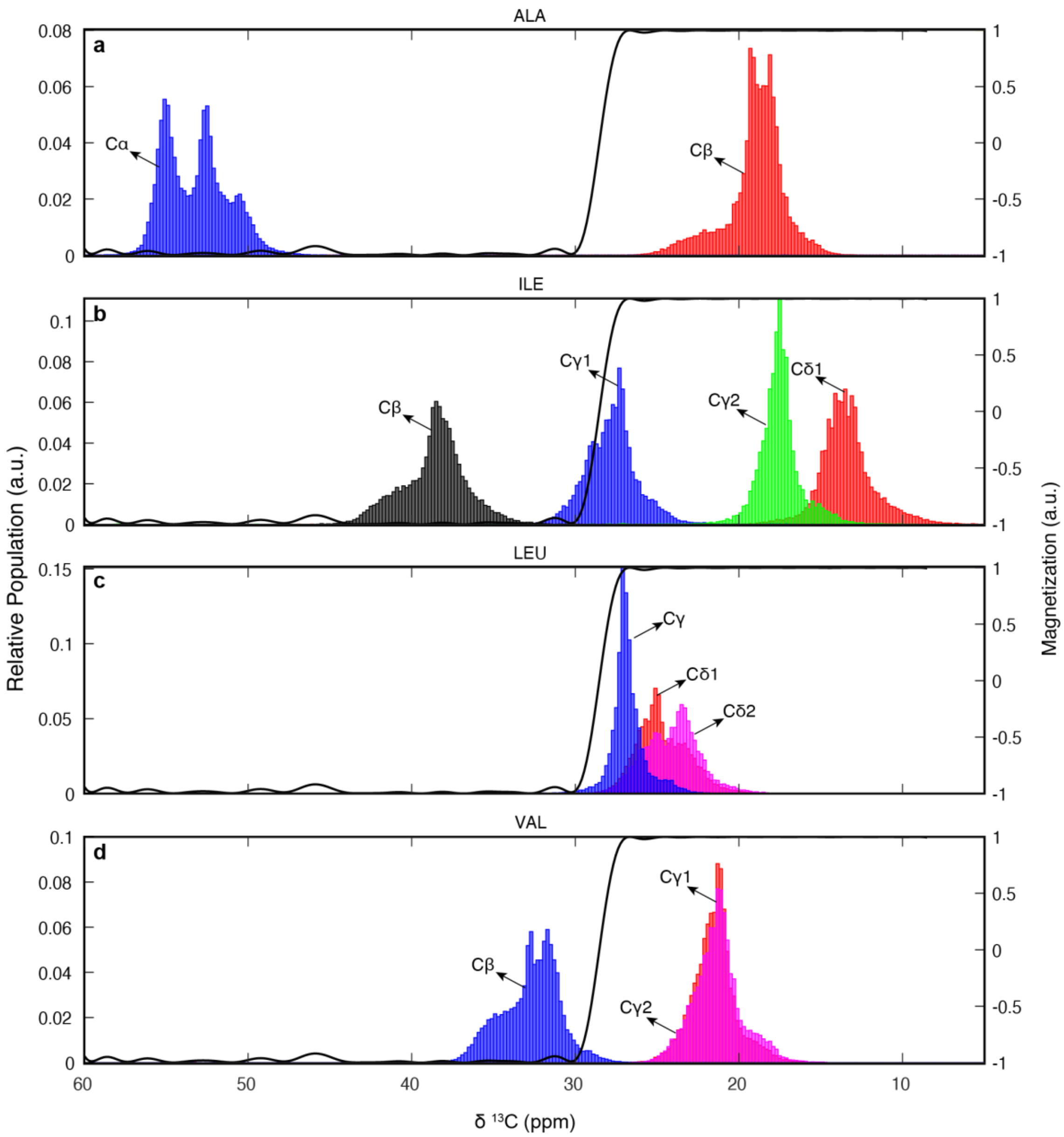

Supplementary Fig. 1: Chemical shift distribution of the methyl and adjacent bonded carbon resonances. The distribution of chemical shifts of the methyl and the directly bonded carbon resonances for amino acids **a.** Ala, **b.** Ile, **c.** Leu and **d.** Val obtained from the BMRB database. The simulated inversion profile under the effect of the selective pulse is shown as line plot (black) assuming an initial magnetization of $+I_z$. It can be inferred from the figure that the $C^{\gamma 1}_{Ile}$ and C^{β}_{Ala} are decoupled from C^{β}_{Ile} and C^{α}_{Ala} respectively during chemical shift evolution by placing the optimally designed selective pulse in middle of the indirect evolution time. The left Y-axis represents the normalized population distribution of the individual atom of the denoted amino acid. The normalization is done with respect to each atom of a given amino acid such that the area under the curve is set to 1.

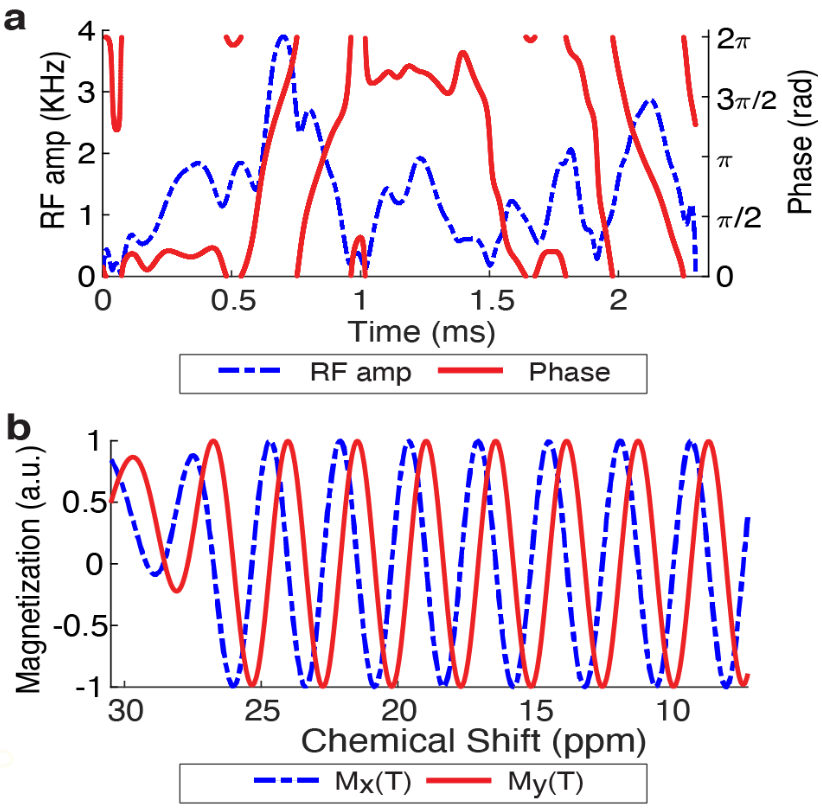

Supplementary Fig. 2: Properties of the valine-selective decoupling pulse designed using optimal control theory. a. The amplitude (blue) and phase profile of the shaped pulse(red) of the selective pulse is shown. The duration of the pulse on an 800 MHz NMR spectrometer is 2.3 ms and the maximum power of the pulse is 3.89 KHz. The mean power of the shape pulse is 1.39 KHz. **b.** Simulated final $\hat{x}$ and $\hat{y}$ magnetization state of the spins after 2.3 ms of pulse duration under the influence of the shaped pulse. The simulation assumes a starting methyl magnetization of I_x. The terminal methyl-peaks are distributed across a signature chemical shift region of 8.5 ppm to 28 ppm, which is sinusoidally encoded during the pulse. This ensures that the inversion of the nearby $C^{\beta}{}_{\mathrm{Val}}$ does not produce Bloch-Siegert shifts or other off-resonance effects on the observed methyl peaks.

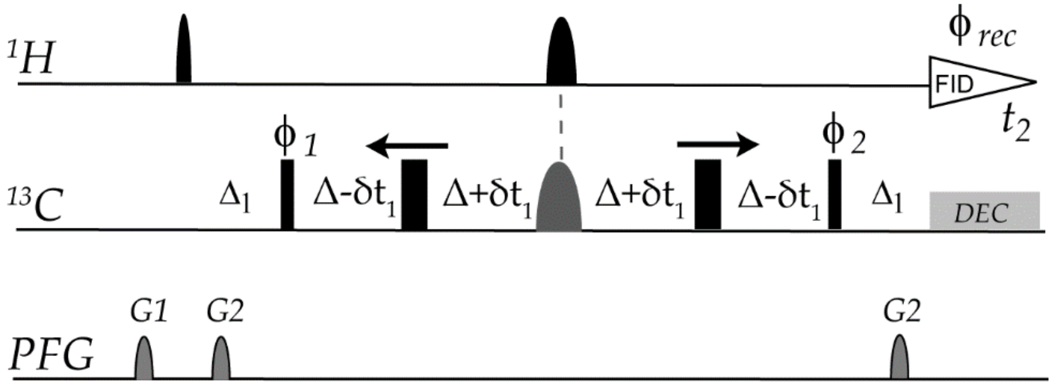

Supplementary Fig. 3: Pulse sequence diagram for constant time SOFAST-HMQC. Narrow and broad rectangular bars represent 90° and 180° pulses, respectively. The ellipsoidal pulses are the shaped pulses. The grey shaded pulse applied in ^{13}C dimension is selective 180° pulse designed using optimal control theory. The phase of the pulse is x unless otherwise indicated. The phase cycle employed are: $\varphi_1 = x, -x$, $\varphi_2 = x, x, -x, -x$ and $\varphi_{rec} = x, -x, -x, x$. The delays used are: $\Delta_1 = 1/(2\ {}^1J_{CH})$ and $\Delta = 1/(4\ {}^1J_{CC})$. The chemical shift in the indirect dimension t_1 is evolved in the constant-time manner. Similar block scheme of constant time encoding of chemical shift is reported in pulse scheme by Törner et.al. [1]. Quadrature detection in the indirect dimension is achieved by incrementing φ_1 using States-TPPI. The gradients G1 and G2 have smoothed squared profile and are applied in the z-direction for a duration of 600 μs, with a recovery delay of 200 μs and a gradient strength 11% and 7% of maximum gradient strength, respectively. In a regular HMQC pulse sequence non-selective rectangular pulses are used in place of the 90° and 180° selective shaped pulses in the ^{1}H channel, and water suppression is achieved by pre-saturation on water resonance during the interscan delay.

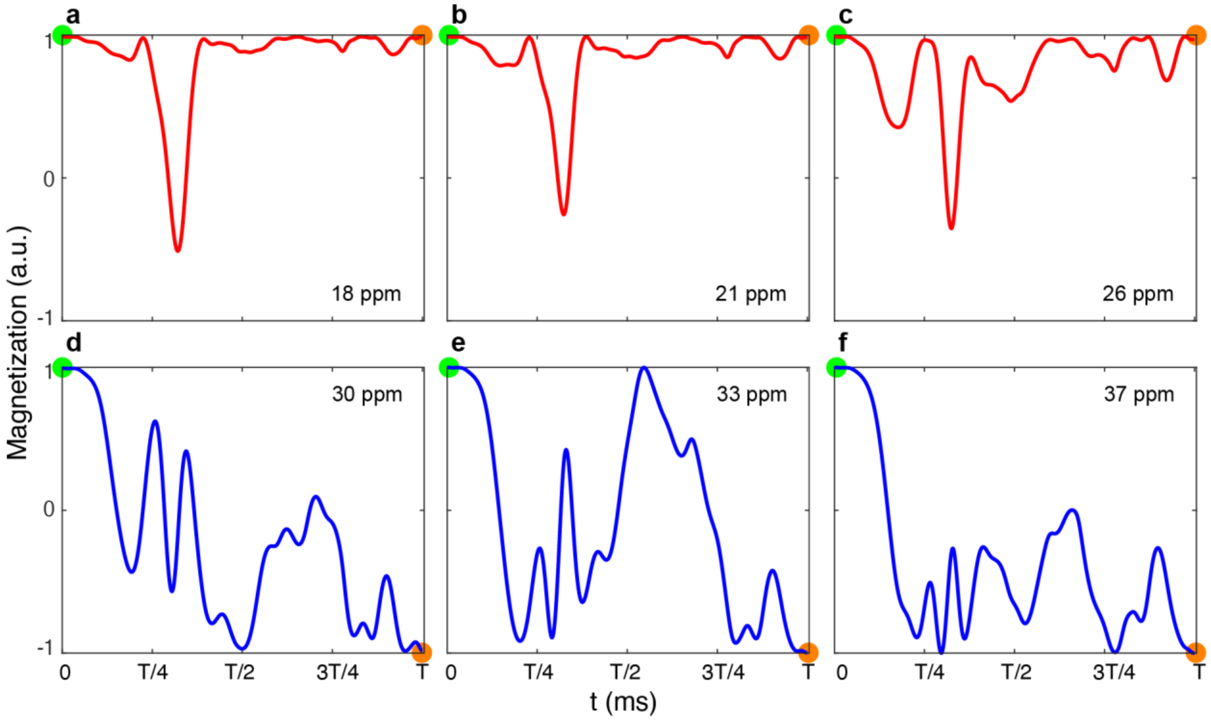

Supplementary Fig. 4: Simulation of the magnetization trajectory for selected chemical shifts under the influence of the shaped pulse. Simulations show the trajectories of longitudinal magnetization, at the frequency corresponding to the chemical shift indicated in the plot, during the duration of the 2.3 ms shape pulse. Although the spins at different frequencies follow different intricate trajectories *during* the pulse, each one reaches its target state exactly at time T = 2.3 ms. The initial state is $+I_z$ and the target state is either $+I_z$ or $-I_z$ depending on the resonance frequency. Green dot indicates the initial magnetization at the start of the shaped pulse and orange dot indicated the state of the magnetization at the end of the shaped pulse. A magnetization that starts at 1 and ends at -1 would indicate inversion. **a, b, c.** For chemical shifts sampled from the C^{γ}_{Leu} chemical shift bandwidth, the initial and target states are both $+I_z$. This ensures the coupling in leucine is *not* refocused. Also, the observed C_{methyl} resonances in the same bandwidth are in the transverse plane at the beginning and the end of the pulse (Supplementary Fig. 1) and their net rotation during the pulse is purely about the $\hat{z}$-axis. A rotation about $\hat{z}$ implies that any $\hat{z}$ -component of magnetization is unchanged, so the initial and target longitudinal magnetization of $+I_z$ is also appropriate for the C_{methyl} resonances. **d, e, f.** For chemical shifts associated with C^{β}_{Val} the target state is instead $-I_z$. Inversion of C^{β}_{Val} is sufficient for decoupling it from the observed valine methyl resonances.

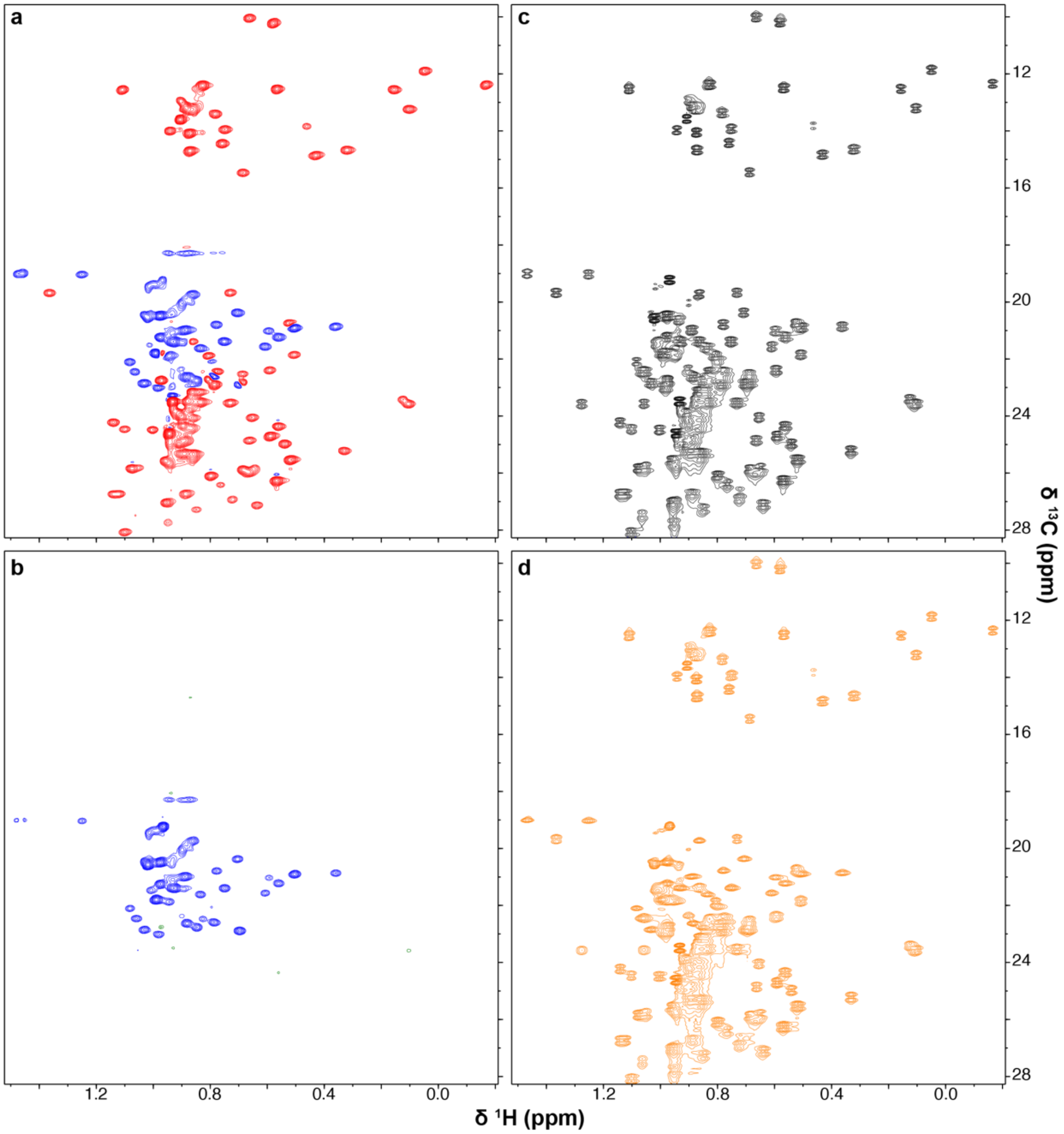

Supplementary Fig. 5: ^{1}H–^{13}C HMQC spectra of MBP with various implementations of the valine specific decoupling pulse. a. constant-time ^{1}H–^{13}C HMQC spectra of MBP with total constant encoding time of $T = 1/{}^{1}J_{CC}$ showing the leucine and isoleucine peaks (red) in opposite phase compared to valine peaks (blue). **b.** With constant-time duration $T = 3/(2\ {}^{1}J_{CC})$ only valine peaks (blue) are present in the spectrum. **c.** Real-time ^{1}H–^{13}C HMQC spectrum of MBP with chemical shift evolved for sufficient time to observe peak doublets caused by scalar coupling $^{1}J_{CC}$. **d.** Real-time ^{1}H–^{13}C HMQC spectrum of MBP in presence of the selective decoupling pulse during the chemical shift encoding duration. There is a clear and selective change in the lineshape of the valine peaks from doublets to singlets in the presence of the selective decoupling pulse.

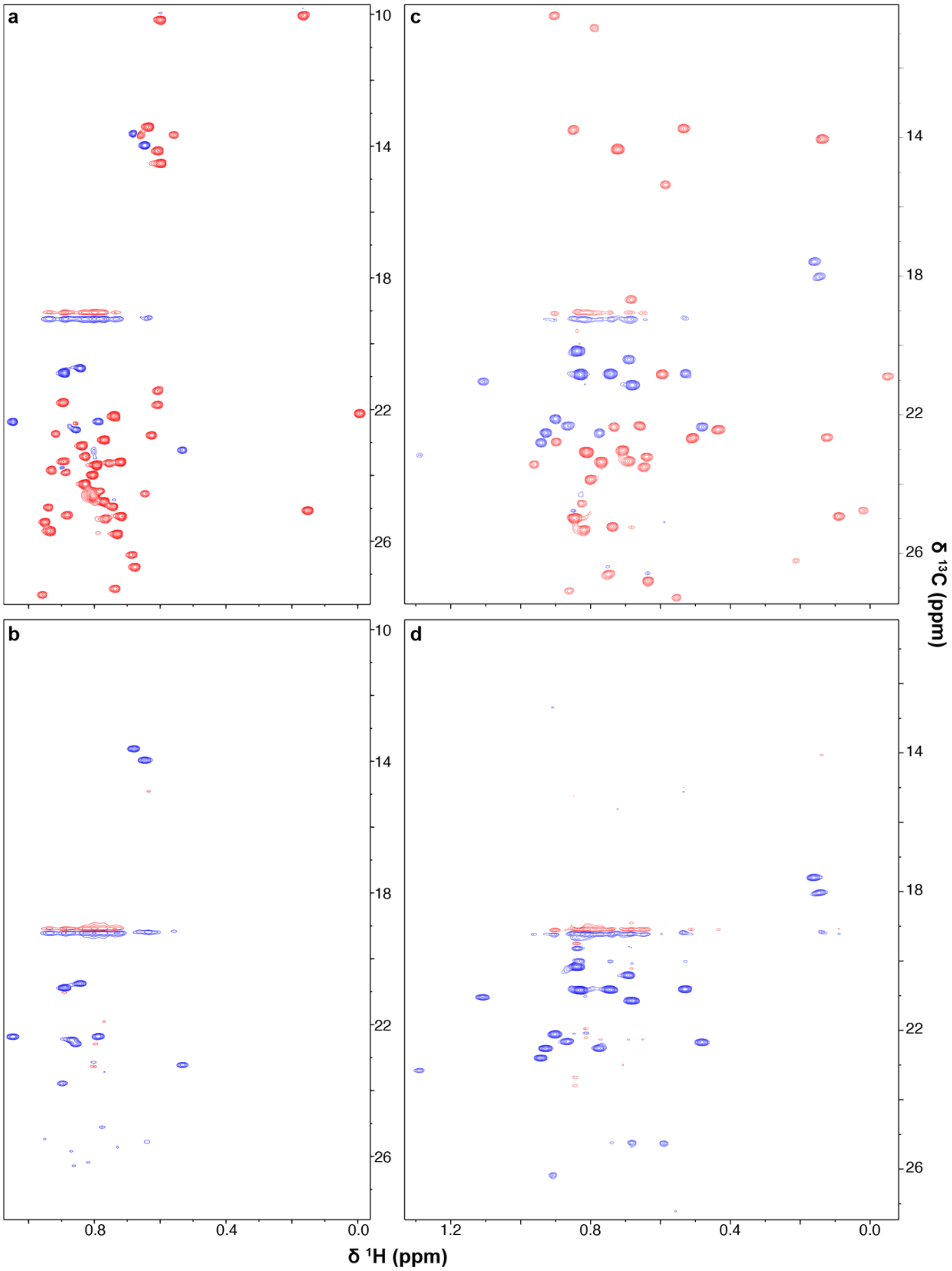

Supplementary Fig. 6: ^{1}H–^{13}C HMQC spectra of human IL-2 and Cas9 HNH domain. Constant-time ^{1}H–^{13}C HMQC spectra of 15.4 kDa human IL-2 (920 μM) using selective decoupling pulse along with constant-time duration of **a.** 28 ms and **b.** 42 ms. Constant-time ^{1}H–^{13}C HMQC spectra of 15 kDa HNH domain of Cas9 (130 μM) using selective decoupling pulse along with constant-time duration of **c.** 28 ms and **d.** 42 ms. The artefact observed at carrier frequency in the ^{13}C dimension are axial peaks, probably arising due to the rapid scanning. Since this region of the spectra is normally empty this does not affect the quality of the spectrum.

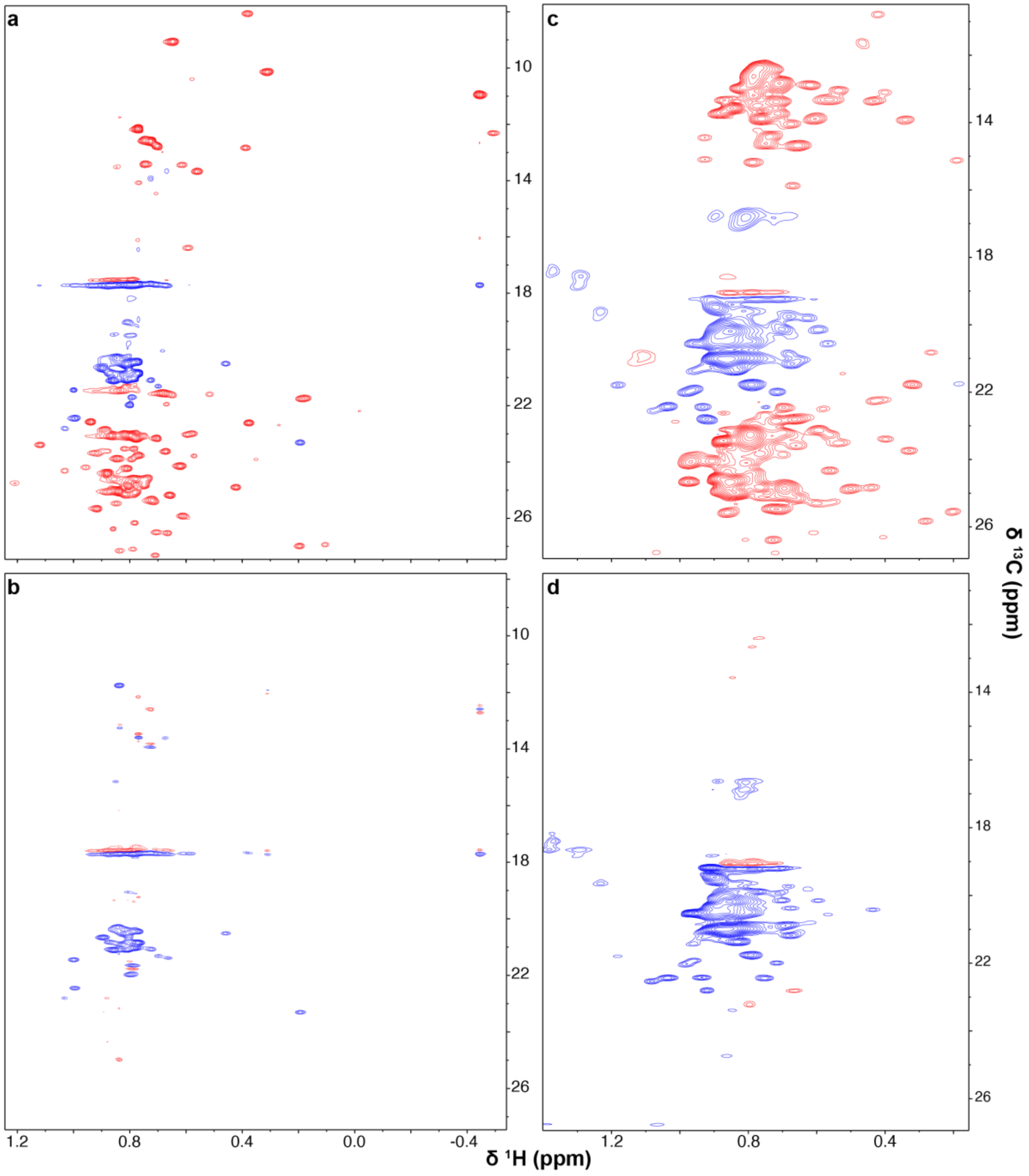

Supplementary Fig. 7: ^{1}H–^{13}C HMQC spectra of Cas9 Rec1-2 domain and eIF4A. Constant-time ^{1}H–^{13}C HMQC spectra of 52 kDa Rec1-2 domain of Cas9 (250 μM) using selective decoupling pulse along with constant-time duration of **a.** 28 ms and **b.** 42 ms. constant-time ^{1}H–^{13}C HMQC spectra of 46 kDa eIF4A (50 μM) using selective decoupling pulse along with constant-time duration of **c.** 28 ms and **d.** 42 ms. The artefact observed at carrier frequency in the ^{13}C dimension are axial peaks, probably arising due to the rapid scanning. Since this region of the spectra is normally empty this does not affect the quality of the spectrum.

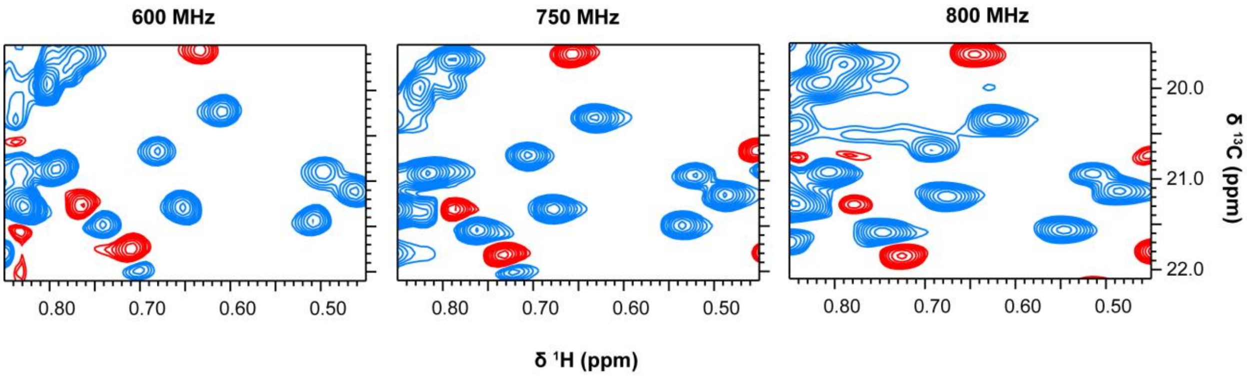

Supplementary Fig. 8: Demonstration of applicability of the pulse at different magnetic field strengths. The pulse created for 800 MHz magnetic field strength was applied to MBP, after a proper scaling of the pulse duration and pulse power, using NMR spectrometers operating at 600 MHz and 750 MHz proton Larmor frequency. The spectra shown are the constant-time HMQC with the constant time period T set to 28 ms for MBP.

Supplementary Table 1. Scaling of pulse duration and pulse power with magnetic field. A user can read in the pulse duration and pulse power from the table below for different magnetic field strengths. We can use the formula $20*log_{10}(\tau_p/\tau)$ to calculate the change in the power level in dB units. Here τ_p is read from the table below and τ is the pulse duration of hard 90 pulse in ^{13}C dimension.

Magnetic Field (MHz)	Pulse Duration (T_p) (μs)	Pulse Power (τ_p) (μs)
**500**	3680	40.0
**600**	3067	33.3
**700**	2629	28.6
**750**	2453	26.7
**800**	2300	25.0
**900**	2044	22.2
**1000**	1840	20.0
**1100**	1673	18.2
**1200**	1533	16.7

Supplementary Table 2. Two dimensional ^{1}H-^{13}C HMQC NMR experiment acquisition parameters for proteins used in this study.

		MBP (real time)	MBP (constant time)	eIF4A	Human IL-2	Cas9 HNH domain	Cas9 REC1-2 domain
**No. of complex points (real +imaginary)**	^{1}H	2048	2048	1024	2048	2048	2048
	^{13}C	512	222	212	238	218	268
**Acquisition time (ms)**	^{1}H	85	85	42	80	80	80
	^{13}C	65	28	28	28	28	28
**Spectral Width (ppm)**	^{1}H	16	16	16	16	16	16
	^{13}C	21	21	20	20	19	22.9
**Carrier Frequency (ppm)**	^{1}H	4.7	4.7	4.7	4.7	4.7	4.7
	^{13}C	15.5	15.5	16.5	16.5	16.5	15
**Spectrometer Frequency (MHz)**		750	750	750	800	800	800
**Temperature (K)**		298	298	298	298	298	298
**No. of scans**		24	24	128	16	24	48
**Interscan delay (sec)**		1	1	1	0.2	0.2	0.2

Supplementary Table 3. Sequences of codon optimized genes for *E. coli* expression.

Protein	Codon optimized genes
**Cas9 HNH domain**	aacagccgtgagcgtatgaagcgtatcgaggaaggtattaaagaactgggcagccagattctgaaggagcacccggtggaaaaca<br>cccagctgcaaaacgagaaactgtacctgtactatctgcagaacggtcgtgatatgtatgttgaccaagaactggatatcaaccgtctg<br>agcgactacgatgtggaccacattgttccgcaaagcttcctgaaggacgatagcatcgacaacaaagtgctgacccgtagcgacaaa<br>aaccgtggcaagagcgataacgttccgagcgaggaagtggttaagaaaatgaagaactattggcgtcagctgctgaacgcgaaact<br>gatcacccagcgtaagtttgataacctgaccaaggcggaacgtggtggcctgggtggcggtagcggtacccgtctgccgaagaaaa<br>agcgtaaagtgggcggtggcagccaccaccaccaccaccac
**Cas9 REC1-2 domain**	ggcgaaaccgcggaagcaacgcgtctgaaacgtaccgcacgtcgccgttacacgcgccgtaaaaatcgtatttgctatctgcagga<br>aatctttagcaacgaaatggcgaaagtcgatgactcatttttccaccgcctggaagaatcgtttctggtggaagaagataaaaaacatg<br>aacgtcacccgattttcggcaatatcgttgatgaagtcgcgtaccatgaaaaatatccgacgatttaccacctgcgtaaaaaactggtgg<br>attctaccgacaaagccgatctgcgcctgatttatctggcactggctcatatgatcaaatttcgtggtcacttcctgattgaaggcgacct<br>gaacccggataatagtgacgtcgataaactgtttattcagctggtgcaaacctataatcagctgttcgaagaaaacccgatcaatgcaa<br>gtggtgttgatgcgaaagccattctgtccgctcgcctgagtaaatcccgccgtctggaaaacctgattgcacagctgccgggtgaaaa<br>gaaaaacggtctgtttggcaatctgatcgctctgtcactgggcctgacgccgaactttaaatcgaatttcgacctggcagaagatgctaa<br>actgcagctgagcaaagatacctacgatgacgatctggacaacctgctggcgcaaattggcgaccagtatgccgacctgtttctggc<br>ggccaaaaatctgtcagatgccattctgctgtcggacatcctgcgcgtgaacaccgaaatcacgaaagcgccgctgtcagcctcgat<br>gattaaacgctacgatgaacatcaccaggacctgaccctgctgaaagcactggttcgtcagcaactgccggaaaaatacaaagaaat<br>tttctttgaccaaagtaaaaatggttatgcaggctacatcgatggcggtgcttcccaggaagaattctacaaattcatcaaaccgatcctg<br>gaaaaaatggatggtacggaagaactgctggtgaaactgaatcgtgaagatctgctgcgtaaacaacgcacctttgacaacggtagc<br>attccgcatcagatccacctgggcgaactgcatgcgattctgcgccgtcaggaagatttttatccgttcctgaaagacaaccgtgaaaa<br>aatcgaaaaaatcctgacgtttcgcatcccgtattacgttggtccgctggcacgtggtaatagccgcttcgcatggatgacccgcaaat<br>ctgaagaaaccattacgccgtggaactttgaagaagtggttgataaaggcgcaagcgctcagtcttttatcgaacgtatgaccaatttcg<br>ataaaaacctgccgaatgaa
**Human IL-2**	atggccccgacaagttcaagcacaaagaagacccagttacaattagagcatcttctgctgganttgcagatgatcttaaacggtattaat<br>aattacaaaaatcccaagttgactcgtatgctgacgtttaaattctatatgccaaaaaaggctacggagcttaaacatctgcaatgcctgg<br>aagaggagcttaaaccgttggaggaagttcttaatcttgcccagagtaagaatttccacttacgtccgcgtgacttgattagtantatcaa<br>tgtgatcgtattggaattgaaaggcagtgagacgacgttcatgtgcgaatatgcagacganacggcgaccatcgtggagttcctgaat<br>cgctggattacgttctcccaatccatcatctccactcttacc

References

1 Törner, R., Awad, R., Gans, P., Brutscher, B. & Boisbouvier, J. Spectral editing of intra- and inter-chain methyl–methyl NOEs in protein complexes. *Journal of Biomolecular NMR* **74**, 83-94 (2020).
