# Supplementary material for: Nearest-neighbor NMR spectroscopy: categorizing spectral peaks by their adjacent nuclei
Source: Nat Commun. 2020 Nov 3;11:5547. doi: 10.1038/s41467-020-19325-4 (PMC7642304; doi:10.1038/s41467-020-19325-4)
Supplement: Supplementary file 2 — Description of Additional Supplementary Files [file 41467_2020_19325_MOESM2_ESM.pdf]

## Description of Additional Supplementary Files

File Name: Supplementary Data 1
Description: Selective homonuclear decoupling pulse to distinguish leucine from valine.

File Name: Supplementary Data 2
Description: HMQC pulse scheme for real time $^{13}C$ chemical shift evolution along with the selective homonuclear decoupling pulse.

File Name: Supplementary Data 3
Description: SOFAST HMQC pulse scheme for constant time $^{13}C$ chemical shift evolution along with the selective homonuclear decoupling pulse.
